# Welche Branchen sind ökonomisch systemrelevant?

**DOI:** 10.1007/s10273-020-2739-7

**Published:** 2020-09-25

**Authors:** Christian Schneemann, Enzo Weber, Marc Ingo Wolter, Gerd Zika

**Affiliations:** 1 Forschungsbereich A1, Inst. f. Arbeitsmarkt- und Berufsforschung, Regensburger Str. 100, 90478 Nürnberg, Deutschland; 2 Bereich Wirtschaft und Soziales, Gesellschaft für Wirtschaftliche Strukturforschung, Heinrichstraße 30, 49080 Osnabrück, Deutschland; 3 Forschungsbereich A2, Inst. f. Arbeitsmarkt- und Berufsforschung, Regensburger Straße 104, 90478 Nürnberg, Deutschland

**Keywords:** C67, D57, E17

## Abstract

Im Zuge der Coronavirus-Pandemie stellt sich die Frage, welche Branchen systemrelevant sind. An erster Stelle stehen Bereiche wie Gesundheit und Pflege sowie alle Branchen, die die Versorgung des täglichen Bedarfs abdecken. Dabei reicht es jedoch nicht, nur die Branchen anzuschauen, die direkt für den Endverbrauch wichtig sind. Die gesamten Wertschöpfungsketten müssen betrachtet werden. Mithilfe der Input-Output-Rechnung werden die Vorleistungen einbezogen und die besonders systemrelevanten Branchen identifiziert.

## Abstract (en)

With the economic shutdown in the wake of the COVID-19 pandemic, the importance of some industries for the national economy has come into focus. This report identifies industries that are closely linked with the rest of the economy through supply chains; stopping production in these industries would have a significant impact on the overall economy. On the one hand, there are industries in which production losses lead to massive demand losses in other industries. On the other hand, there are industries in which production downtimes lead to massive delivery difficulties for other industries. Beyond these interdependencies, we also account for negative effects stemming from the loss of income and from consumption or investment decisions.

Das Coronavirus trifft die Länder Europas unvermittelt und hart. Um die Ausbreitung des Virus zu bremsen, entschlossen sich die Regierungen Europas zum „shut down". Schulen, Kitas, Universitäten sowie öffentliche Einrichtungen jeder Art wurden geschlossen. Veranstaltungen wurden unterbunden und nur Geschäfte, die als gesellschaftlich notwendig gelten (z.B. Einzelhandel mit Lebensmitteln), durften geöffnet bleiben. Momentan sind viele Bereiche der Wirtschaft unter Auflagen in einer „neuen Normalität" angekommen. Zudem wurden Konjunkturpakete geschnürt, um mit unterschiedlichen wirtschaftlichen Impulsen die Wirtschaft zu stabilisieren. Um diese Impulse gezielt setzen zu können, stellt sich die Frage, welche Bereiche des öffentlichen Lebens bzw. welche Branchen[1] systemrelevant sind. Unbestritten leisten Bereiche wie Gesundheit und Pflege einen wichtigen gesellschaftlichen Beitrag, gerade in der aktuellen COVID-19-Pandemie. Unverzichtbar sind auch alle Branchen, die im Zusammenhang mit der Versorgung des täglichen Bedarfs stehen, wie der Lebensmittel-Einzelhandel oder die Nahrungsmittelindustrie. Hier geht es also um Branchen in Deutschland, deren Produkte für den Endverbrauch unmittelbar besonders relevant sind.

Doch welche Branchen sind aufgrund der dort produzierten Vorleistungen besonders eng über die Wertschöpfungsketten mit der übrigen Wirtschaft verflochten oder haben besonders großen Einfluss über Kreislaufeffekte – sodass ein Produktionsstopp in diesen Bereichen die Gesamtwirtschaft besonders stark in Mitleidenschaft zieht?

Hierzu wird in einem ersten Schritt die Input-Output-Tabelle (IOT) des Statistischen Bundesamts (2010) ausgewertet. Die Input-Output-Rechnung eignet sich besonders gut für diese Art der Analyse, da diese zum Ziel hat, Verflechtungen innerhalb einer Volkswirtschaft und mit

1 In den Volkswirtschaftlichen Gesamtrechnungen werden je nach Ausrichtung der Fachstatistik zwischen den Begriffen Wirtschaftszweig, Produktionsbereich und Gütergruppe unterschieden. Im Folgenden verwenden wir einheitlich zur Vereinfachung den Begriff Branche, der die größten Überschneidungen zwischen den genannten Begriffen hat.

**Christian Schneemann** ist Mitarbeiter im Forschungsbereich „Prognosen und gesamtwirtschaftliche Analysen" am Institut für Arbeitsmarkt- und Berufsforschung (IAB).

**Prof. Dr. Enzo Weber** ist Leiter des Forschungsbereichs „Prognosen und gesamtwirtschaftliche Analysen" am IAB und Professor für Empirische Wirtschaftsforschung, insbesondere Makroökonometrie und Arbeitsmarkt, an der Universität Regensburg.

**Dr. Gerd Zika** ist wissenschaftlicher Mitarbeiter im Forschungsbereich „Prognosen und Strukturanalysen" sowie in der Arbeitsgruppe „Digitale und ökologische Transformation" am IAB.

der übrigen Welt darzustellen. So werden direkte Wirkungen (Produktionsausfälle) um die indirekten Wirkungen, also solche auf andere Branchen, ergänzt. In einem zweiten Schritt werden zudem induzierte Effekte (Lohn-Einkommen-Konsum, Investitionsentscheidungen) eines Produktionsrückgangs der identifizierten Branchen mit Hilfe eines makroökonomischen Modells analysiert. Dafür wird auf das Modellinstrumentarium zurückgegriffen, dass bereits bei den BIBB-IAB-Qualifikations- und Berufsprojektionen zum Einsatz kommt (Maier et al., 2018).

## Identifikation systemrelevanter Branchen

Um die ökonomisch systemrelevanten Branchen zu identifizieren, werden spezifische Indikatoren analysiert, die mit der IOT 2016 berechnet wurden:

- *Indikator 1*: Anteil der in der Branche erzielten Bruttowertschöpfung bezogen auf die Bruttowertschöpfung insgesamt (direkte Wirkung). Die Bruttowertschöpfung ist Teil der Volkswirtschaftlichen Gesamtrechnungen und ergibt sich aus dem Gesamtwert der im Produktionsprozess erzeugten Waren und Dienstleistungen (Produktionswert), abzüglich des Werts der Vorleistungen. Sie entspricht abzüglich der erhaltenen Gütersubventionen dem Bruttoinlandsprodukt (BIP). Daraus lässt sich also ablesen, wie viel Prozent die einzelnen Branchen zur Wirtschaftsleistung der Gesamtwirtschaft beitragen. Allerdings kann der Indikator nicht so interpretiert werden, dass das BIP im Jahr 2016 um den ausgewiesenen Prozentsatz einer Branche niedriger ausgefallen wäre, wenn in dieser Branche im ganzen Jahr keine Güter bzw. Dienstleistungen produziert worden wären. Für eine solche Interpretation greift der Indikator zu kurz, weil die Vorleistungen, die eine Branche bezieht, nicht berücksichtigt werden. Würden in der spezifischen Branche keine Güter bzw. Dienstleistungen hergestellt werden, so würden die hierfür benötigten Vorleistungen ebenfalls nicht nachgefragt und dementsprechend nicht produziert werden.

- *Indikator 2*: Anteil der von der Branche bezogenen Vorleistungen (nur Bruttowertschöpfungsanteil) bezogen auf die Bruttowertschöpfung insgesamt (indirekte Wirkung). Daher gibt der zweite Indikator Aufschluss darüber, welche Branchen besonders viele Vorleistungen beziehen und demnach bei einem Produktionsausfall auch viele andere in Mitleidenschaft ziehen würden. Dieser Indikator gibt jedoch nicht nur Aufschluss darüber, wie viel Nachfrageausfall bei anderen Branchen der Produktionsausfall einer spezifischen Branche nach sich zieht. Umgekehrt ist dieser Indikator auch ein Indiz, wie stark die spezifische Branche von anderen Branchen über die benötigten Vorleistungen abhängt. Je höher der prozentuale Anteil, desto wichtiger sind andere Branchen für die eigene Bruttowertschöpfung.

- *Indikator 3*: Anteil der von der Branche gelieferten Vorleistungen (ohne interne) bezogen auf die gesamten Vorleistungen. Der dritte Indikator beleuchtet die Bedeutung einer Branche für die inländische Produktion. Er gibt an, welcher prozentuale Anteil der produzierten Waren und Dienstleistungen in anderen Branchen weiterverarbeitet wird. Je höher dieser Anteil ist, desto weitreichender sind die Folgen eines Produktionsausfalls für die anderen Bereiche. Die bislang genannten Indikatoren werden von der Größe der jeweiligen Branche bezogen auf den Umfang der dort erbrachten Wirtschaftsleistung beeinflusst: Eine große Branche liefert und bezieht in der Summe tendenziell mehr Vorleistungen als eine kleine Branche. Daher bilden die beiden folgenden Indikatoren die Verflechtungen zwischen den Branchen unabhängig von ihrer Größe ab.

- *Indikator 4*: Anteil der von der Branche bezogenen Vorleistungen (nur Bruttowertschöpfungsanteil) bezogen auf den Produktionswert der Branche. Der vierte Indikator gibt Aufschluss darüber, welche Branchen besonders viele Vorleistungen beziehen und demnach bei einem Produktionsausfall auch viele andere in Mitleidenschaft ziehen würden. Zudem ist dieser Indikator auch ein Indiz, wie stark die spezifische Branche von anderen Branchen über die benötigten Vorleistungen abhängt. Je höher der prozentuale Anteil, desto wichtiger sind andere Branchen für diese Branche und desto kleiner ist die in der Branche selbst erbrachte Wertschöpfung.

- *Indikator 5*: Anteil der an andere Branchen gelieferten Vorleistungen bezogen auf den Produktionswert der Branche. Der fünfte Indikator beleuchtet die Bedeutung einer Branche für die inländische Produktion. Er gibt an, welcher prozentuale Anteil der produzierten Waren und Dienstleistungen in anderen Branchen weiterverarbeitet wird. Je höher dieser Anteil ist, desto weitreichender sind die Folgen eines Produktionsausfalls für die anderen Bereiche und desto größer ist die Abhängigkeit dieser Branche von der Auftragslage der anderen Branchen.

Tabelle 1 enthält eine Rangfolge für die Indikatoren 1 bis 3 sowie das als Rang insgesamt bezeichnete arithmetische Mittel der Einzelränge. Auffällig ist, dass unter den Top 20 der Branchen mit Metallerzeugnisse (Rang 11), Kraftwagen und Kraftwagenteile (Rang 12), Nahrungs- und Futtermittel, Getränke, Tabakerzeugnisse (Rang 13) sowie Maschinen (Rang 14) nur vier Branchen sind, die Waren herstellen. Unter den Top 10 sind sogar ausschließlich

Tabelle 1
**Indikatoren 1 bis 3 zur Identifikation systemrelevanter Branchen: Ränge 1 bis 20**

| Branche | Bruttowertschöpfung der Branche bezogen auf die gesamte Bruttowertschöpfung | | Von anderen Branchen bezogene Vorleistungen (nur Bruttowertschöpfung) bezogen auf die gesamte Bruttowertschöpfung | | An andere Branchen gelieferte Vorleistungen bezogen auf alle Vorleistungen | | Rang insgesamt |
|---|---|---|---|---|---|---|---|
| | in % | Rang | in % | Rang | in % | Rang | |
| Dienstleistungen des Grundstücks- und Wohnungswesens | 11,54 | 1 | 1,44 | 4 | 7,99 | 1 | 1 |
| Großhandelsleistungen (ohne Handelsleistungen mit Kfz) | 5,19 | 4 | 1,55 | 2 | 6,17 | 2 | 2 |
| Vorbereitende Baustellen-, Bauinstallations- und sonstige Ausbauarbeiten | 3,47 | 8 | 1,26 | 6 | 4,98 | 4 | 3 |
| Einzelhandelsleistungen (ohne Handelsleistungen mit Kfz) | 3,68 | 7 | 1,50 | 3 | 3,17 | 10 | 4 |
| Dienstleistungen der Rechts-, Steuer- und Unternehmensberatung | 2,91 | 9 | 0,70 | 14 | 5,95 | 3 | 5 |
| Dienstleistungen der öffentlichen Verwaltung und der Verteidigung | 5,20 | 3 | 1,21 | 7 | 2,09 | 16 | 6 |
| Finanzdienstleistungen | 2,54 | 12 | 0,85 | 9 | 3,87 | 7 | 7 |
| Wach-, Sicherheitsdienstleistungen, wirtschaftliche Dienstleistungen | 2,26 | 14 | 0,69 | 15 | 4,81 | 6 | 8 |
| Lagereileistungen, sonstige Dienstleistungen für den Verkehr | 1,58 | 21 | 0,70 | 13 | 4,87 | 5 | 9 |
| Landverkehrs- und Transportleistungen in Rohrfernleitungen | 1,72 | 19 | 0,59 | 18 | 3,49 | 8 | 10 |
| Metallerzeugnisse | 1,91 | 18 | 0,51 | 21 | 2,94 | 11 | 11 |
| Kraftwagen und Kraftwagenteile | 3,69 | 6 | 1,94 | 1 | 0,52 | 43 | 12 |
| Nahrungs- und Futtermittel, Getränke, Tabakerzeugnisse | 1,48 | 23 | 1,29 | 5 | 1,24 | 27 | 13 |
| Maschinen | 2,90 | 10 | 1,17 | 8 | 0,77 | 38 | 14 |
| Dienstleistungen von Architektur- und Ingenieurbüros und der technischen, physikalischen Untersuchung | 1,50 | 22 | 0,51 | 20 | 2,07 | 17 | 15 |
| Elektrischer Strom, Dienstleistungen der Elektrizitäts-, Wärme- und Kälteversorgung | 0,99 | 29 | 0,62 | 17 | 2,38 | 14 | 16 |
| Handelsleistungen mit Kfz, Instandhaltung und Reparatur an Kfz | 2,07 | 16 | 0,38 | 29 | 2,19 | 15 | 17 |
| Dienstleistungen des Gesundheitswesens | 5,27 | 2 | 0,84 | 10 | 0,21 | 56 | 18 |
| IT- und Informationsdienstleistungen | 2,73 | 11 | 0,17 | 49 | 3,34 | 9 | 19 |
| Dienstleistungen von Versicherungen und Pensionskassen | 0,88 | 33 | 0,73 | 11 | 1,38 | 26 | 20 |

Quelle: eigene Darstellung.

Dienstleistungsbereiche zu finden. Diese 20 Branchen können in Bezug auf ihre wirtschaftlichen Verflechtungen und ihren Beitrag zur gesamten Bruttowertschöpfung als besonders systemrelevant bezeichnet werden.

Die hohe Systemrelevanz der Dienstleistungen des Grundstücks- und Wohnungswesens (Rang 1) und der Dienstleistungen der Rechts-, Steuer- und Unternehmensberatung (Rang 5) sind auch das Resultat des Outsourcing-Trends der letzten Jahrzehnte, also der Verlagerung von Tätigkeiten oder Unternehmensteilen aus Produktions- in Dienstleistungsbereiche. Von diesem Trend dürften auch die Wach-, Sicherheitsdienstleistungen, wirtschaftliche Dienstleistungen (Rang 8) profitiert haben. Die hohe Wertschöpfung in der Branche Dienstleistungen des Grundstücks- und Wohnungswesens erklärt sich dadurch, dass hier aus statistischen Gründen neben den gewerblichen Vermietungen auch die gesamte Wohnungsvermietung einschließlich des eigengenutzten Wohnraums als Produktionsleistung verbucht wird. Hinzu kommt, dass diese Branche bezogen auf die Größe der Branche sehr wenig Vorleistungen bezieht. Rang 3, also die vorbereitenden Baustellen-, Bauinstallations- und sonstige Ausbauarbeiten und die Dienstleistung von Architektur- und Ingenieurbüros und den technisch physikalischen Untersuchungen (Rang 15) dürften vom starken Bauboom der letzten Jahre profitiert haben.

Die Topplatzierung für die Großhandelsleistungen (ohne Handelsleistungen mit Kfz) und Einzelhandelsleistungen (ohne Handelsleistungen mit Kfz) mit Rang 2 und 4 ist ein Ausdruck für die hohe Bedeutung des Handels für die Zir-

Tabelle 2

**Indikatoren 4 und 5 zur Identifikation systemrelevanter Branchen: Ränge 1 bis 20**

| | Von anderen Branchen bezogene Vorleistungen (nur Bruttowertschöpfung) bezogen auf die Produktion der Branche | | An andere Branchen gelieferte Vorleistungen (ohne interne) bezogen auf die Produktion der Branche | | Rang insgesamt |
|---|---|---|---|---|---|
| Branche | in % | Rang | in % | Rang | |
| Kohle | 22,07 | 7 | 92,59 | 2 | 1 |
| Sonstige freiberufliche, wissenschaftliche, technische und veterinärmedizinische Dienstleistungen | 23,68 | 5 | 77,44 | 6 | 2 |
| Post-, Kurier- und Expressdienstleistungen | 20,66 | 11 | 87,33 | 4 | 3 |
| Werbe- und Marktforschungsleistungen | 21,92 | 8 | 72,67 | 11 | 4 |
| Mit Finanz- und Versicherungsdienstleistungen verbundene Dienstleistungen | 18,16 | 18 | 76,79 | 7 | 5 |
| Reparatur, Instandhaltung und Installation von Maschinen und Ausrüstungen | 20,69 | 10 | 56,49 | 16 | 6 |
| Wach-, Sicherheitsdienstleistungen, wirtschaftliche Dienstleistungen | 17,68 | 20 | 76,13 | 9 | 7 |
| Erdöl und Erdgas | 17,75 | 19 | 73,25 | 10 | 8 |
| Keramik, bearbeitete Steine und Erden | 20,06 | 13 | 56,26 | 17 | 9 |
| Erze, Steine und Erden, sonstige Bergbauerzeugnisse und Dienstleistungen | 16,85 | 27 | 76,43 | 8 | 10 |
| Druckereileistungen, bespielte Ton-, Bild- und Datenträger | 16,42 | 31 | 77,84 | 5 | 11 |
| Erzeugnisse der Landwirtschaft, Jagd und Dienstleistungen | 19,36 | 16 | 51,41 | 20 | 12 |
| Dienstleistungen von Architektur- und Ingenieurbüros und der technischen, physikalischen Untersuchung | 19,44 | 15 | 48,84 | 22 | 13 |
| Gießereierzeugnisse | 14,90 | 39 | 93,47 | 1 | 14 |
| Dienstleistungen des Verlagswesen | 25,63 | 4 | 35,82 | 36 | 15 |
| Dienstleistungen von Versicherungen und Pensionskassen | 26,44 | 3 | 31,25 | 39 | 16 |
| Dienstleistungen der Abwasser-, Abfallentsorgung und Rückgewinnung | 17,67 | 21 | 47,07 | 23 | 17 |
| Landverkehrs- und Transportleistungen in Rohrfernleitungen | 16,69 | 30 | 60,94 | 15 | 18 |
| Holz, Holz-, Flecht-, Korb- und Korkwaren (ohne Möbel) | 20,51 | 12 | 36,49 | 35 | 19 |
| Tiefbauarbeiten | 26,57 | 2 | 21,44 | 45 | 20 |

Quelle: eigene Darstellung.

kulation der Güter von Produzenten zu Endverbrauchern. Dies wird auch dadurch belegt, dass sich mit den Lagereileistungen, sonstige Dienstleistungen für den Verkehr (Rang 9), den Landverkehrs- und Transportleistungen in Rohrfernleitungen (Rang 10), den Handelsleistungen mit Kfz, Instandhaltung und Reparatur an Kfz (Rang 17) drei weitere Branchen unter den Top 20 befinden, die mit der Bündelung und Verteilung von Warenströmen Geld verdienen.

In Tabelle 2 ist eine Rangfolge für die Indikatoren vier und fünf sowie der Rang insgesamt abgebildet. Auffällig ist, dass unter den Top 20 der Branchen mit Rang 1 Kohle, sowie Erdöl und Erdgas (Rang 8), Keramik, bearbeitete Steine und Erden (Rang 9), Erzeugnisse der Landwirtschaft, Jagd und Dienstleistungen (Rang 12) und Holz, Holz-, Flecht-, Korb- und Korkwaren (ohne Möbel) (Rang 19) Branchen vertreten sind, die Rohstoffe für andere Branchen liefern. Dies liegt vor allem daran, dass hier unabhängig von der Größe der eigenen Branche die Relevanz der Vorleistungen ermittelt wurde. Interessanterweise sind bei dieser Betrachtungsweise mit den sonstigen, freiberuflichen, wissenschaftlichen, technischen und veterinärmedizinischen Dienstleistungen, den mit Finanz- und Versicherungsdienstleistungen verbundene Dienstleistungen sowie den Druckereileistungen, bespielte Ton-, Bild- und Datenträger nun solche Dienstleistungsbranchen auf den vorderen Rängen vertreten, die – bezogen auf die erzielte Bruttowertschöpfung – eher zu den kleinen Branchen zählen. Neben diesen Branchen sind auch hier, genau wie in Tabelle 1, mit der Branche Post-, Kurier- und Expressdienstleistungen (Rang 3) auf den vorderen Plätzen zu finden, die mit der Bündelung und Verteilung von Warenströmen Geld verdient. Und schließlich ist auch mit den Tiefbauarbeiten (Rang 20) das Baugewerbe unter den Top 20 vertreten.

In beiden Tabellen aufgeführt werden nur die Wach-, Sicherheitsdienstleistungen, wirtschaftliche Dienstleistungen, die Dienstleistungen von Architektur- und Ingenieurbüros und der technischen, physikalischen Untersuchung, die Dienstleistungen von Versicherungen und Pensionskassen sowie die Landverkehrs- und Transportleistungen in Rohrfernleitungen. Diese vier Branchen zählen also unabhängig von der Betrachtungsweise zu den Top 20 der ökonomisch systemrelevantesten Branchen.

Zu beachten ist, dass mit dieser Analyse zwar die direkten und die indirekten Produktionswirkungen (Wirkungen auf andere Branchen) betrachtet wurden. Bei einer dynamischen Betrachtung würden jedoch zudem induzierte Wirkungen hinzukommen, die über den Einkommenskreislauf und/oder das Investitionsverhalten vermittelt werden. Mit einem Produktionsausfall sinkt die Arbeitskräftenachfrage, und damit nehmen die Lohneinkommen ab. Dadurch hätten die privaten Haushalte ein geringeres verfügbares Einkommen, sodass auch der private Konsum beeinträchtigt würde, was mittelbar zu einer geringeren Produktion in anderen Branchen führt. Auf der Unternehmensseite führt der Produktionsausfall, aber auch der geringere private Konsum, zu pessimistischeren Erwartungen, was sich negativ auf die Investitionen auswirkt.

Gleichzeitig steigt die Erwerbslosigkeit, was zu einer größeren Belastung der Sozialkassen führt. Dadurch müssen die Sozialbeiträge steigen, wodurch die Lohnnebenkosten zunehmen würden. Alternativ könnte der Staat die Mehrkosten übernehmen, was entweder mit Steuererhöhungen, Schuldenfinanzierung oder Budgetumschichtungen, also beispielsweise der Kürzung von Investitionen, einhergehen müsste.

Schließlich könnte es auch zu Preisreaktionen kommen. Einerseits könnten die Stückkosten und damit die Herstellerpreise für die Güter/Dienstleistungen steigen, die nun nicht mehr im bisherigen Umfang produziert werden. Dies würde zu höheren Kosten und Preisen bei den Unternehmen führen, die diese Güter/Dienstleistungen weiterverarbeiten. Auf der anderen Seite könnte es aber auch zu sinkenden Verkaufspreisen für die Güter/Dienstleistungen kommen, deren Märkte zumindest vorübergehend wegbrechen.

## Gesamtwirtschaftliche Effekte von Produktionsausfällen einzelner Branchen

Um die beschriebenen ökonomischen Folgen von Produktionsausfällen systematisch zu untersuchen, wird das makroökonomischen Modell QINFORGE, das bereits bei den BIBB-IAB-Qualifikations- und Berufsprojektionen[2] zum Einsatz kam, genutzt (Maier et al., 2018). Das Modell erlaubt es, gezielt in die einzelnen Branchen einzugreifen und sowohl die Effekte innerhalb der Branchen, wie auch Effekte auf die Gesamtwirtschaft zu analysieren. Die Eingriffe erfolgten in den Branchen, die den Top 20 Branchen zugeordnet werden. Im Modellrahmen ergeben sich dann gesamtwirtschaftliche Effekte über die oben diskutierten Wirkungskanäle.

In Tabelle 3 werden die Wirkungen auf ausgewählte makroökonomische Kenngrößen dargestellt, wenn es in der jeweiligen Branche zu einem Produktionsausfall kommt, der eine um 1 Mrd. Euro niedrigere Bruttowertschöpfung in dieser Branche zur Folge hat. Die Kenngrößen wurden dahingehend ausgewählt, dass die beschriebenen Kreislaufwirkungen leichter nachvollziehbar sind. Ein Vergleich der beiden Spalten zu den Wirkungen auf die Bruttowertschöpfung der Gesamtwirtschaft zeigt, dass die Kreislaufeffekte im Durchschnitt die isoliert betrachteten Wirkungen aus der Input-Output-Analyse um das Dreifache übersteigen. Besonders große Effekte ergeben sich bei den sonstigen Unternehmensdienstleistungen. In dieser Branche führen Produktionsausfälle zu relativ starken Preisreaktionen aufgrund zunehmender Stückkosten, da die dort erbrachten Leistungen für alle anderen Branchen kurzfristig nur schwer substituierbar sind, können diese Preissteigerungen zum Teil an die Kunden (Leistung beziehenden Branchen) überwälzt werden. Die höheren Preise führen insgesamt zu relativ hohen Rückgängen bei den getätigten Investitionen – die preisbereinigte Produktion und somit auch die Bruttowertschöpfung sinkt stark.

Eine hohe ökonomische Systemrelevanz haben neben den sonstigen Unternehmensdienstleistungen auch die Erbringung von Finanzdienstleistungen, die Landverkehrs- und Transportleistungen in Rohrfernleitungen, der Einzelhandel (ohne Handel mit Kraftfahrzeugen) sowie die Branche Nahrungs- und Futtermittel, Getränke, Tabakerzeugnisse. Bei den Branchen Einzelhandel (ohne Handel mit Kraftfahrzeugen) und Landverkehrs- und Transportleistungen in Rohrfernleitungen erklärt sich die hohe Systemrelevanz vor allem über die starken Konsumausfälle.

Bezogen auf die Zahl der Erwerbstätigen in der jeweiligen Branche zeigt sich, dass die Produktion in den Branchen Gastgewerbe und Dienstleistungen der Kunst und Kultur, Glücksspiel besonders beschäftigungsintensiv ist, denn dort wird die höchste Zahl an Erwerbstätigen durch den Produktionsausfall freigestellt. Aber auch die Herstellung von Nahrungs- und Futtermittel, Getränke, Tabakerzeug-

2 Siehe auch BIBB-IAB-Qualifikations- und Berufsprojektionen, www.QuBe-Projekt.de (17. August 2020).

Tabelle 3
**Kreislaufwirkungen eines Produktionsausfalls (-1 Mrd. Euro Bruttowertschöpfung) ausgewählter Branchen**

| | Input-Output-Tabelle | Kreislaufmodell | | | | |
|---|---|---|---|---|---|---|
| | Bruttowertschöpfung Gesamtwirtschaft | Erwerbstätige in der Branche | Konsum Gesamtwirtschaft | Investitionen Gesamtwirtschaft | Bruttowertschöpfung Gesamtwirtschaft | Erwerbstätige Gesamtwirtschaft |
| | in Mrd. Euro | in 1000 Personen | in Mrd. Euro | in Mrd. Euro | in Mrd. Euro | in 1000 Personen |
| Nahrungs- und Futtermittel, Getränke, Tabakerzeugnisse | -1,87 | -12,5 | -2,2 | -1,8 | -5,5 | -29,8 |
| Herstellung von chemischen Erzeugnissen | -1,46 | -2,6 | -1 | -1,1 | -4 | -13,3 |
| Herstellung von Metallerzeugnissen | -1,26 | -5,6 | -0,7 | -0,8 | -3,1 | -13 |
| Maschinenbau | -1,4 | -4,4 | -0,6 | -0,7 | -2,7 | -11,3 |
| Herstellung von Kraftwagen und Kraftwagenteilen | -1,54 | -1,3 | -1,5 | -0,8 | -3 | -9,8 |
| Baugewerbe | -1,47 | -7,7 | -0,9 | -3,3 | -2,8 | -15,4 |
| Großhandel (ohne Handel mit Kraftfahrzeugen) | -1,3 | -1 | -0,6 | -0,7 | -2,3 | -8,5 |
| Einzelhandel (ohne Handel mit Kraftfahrzeugen) | -1,41 | -10,5 | -10,3 | -1,6 | -6,5 | -29,6 |
| Landverkehrs- und Transportleistungen in Rohrfernleitungen | -1,35 | -9,4 | -7,8 | -2,4 | -6,7 | -28,1 |
| Lagereileistungen, sonstige Dienstleistungen für den Verkehr | -1,45 | -6,4 | -0,7 | -1,3 | -3,5 | -15,7 |
| Gastgewerbe | -1,44 | -23,4 | -3,5 | -0,8 | -3 | -31,4 |
| Erbringung von Finanzdienstleistungen | -1,36 | -12,8 | -2,2 | -1,7 | -8,1 | -27,7 |
| Grundstücks- und Wohnungswesen | -1,13 | -0,4 | -1,4 | -0,2 | -1,5 | -2,4 |
| Dienstleistungen der Rechts-, Steuer- und Unternehmensberatung | -1,24 | -2,5 | -0,4 | -0,3 | -2,2 | -6,4 |
| Sonstige Unternehmensdienstleistungen | -1,31 | -2,4 | -2,8 | -2,5 | -13,1 | -35,3 |
| Öffentliche Verwaltung, Verteidigung, Sozialversicherung | -1,23 | -14,4 | -2,2 | -0,5 | -2,1 | -18 |
| Dienstleistungen der Kunst, der Kultur und des Glücksspiels | -1,17 | -21,1 | -4 | -0,8 | -3 | -35,1 |
| Erbringung von Dienstleistungen des Sports, der Unterhaltung und der Erholung | -1,16 | -8,3 | -2,9 | -0,8 | -3 | -13,9 |
| Erbringung von sonstigen überwiegend persönlichen Dienstleistungen | -1,13 | -8,1 | -0,9 | -0,8 | -3 | -13,5 |

Quelle: eigene Darstellung.

nisse sowie die Erbringung von Finanzdienstleistungen ist relativ arbeitsintensiv. Die größten gesamtwirtschaftlichen negativen Beschäftigungseffekte haben bei einem Produktionsausfall die sonstigen Unternehmensdienstleistungen, dicht gefolgt von den Dienstleistungen der Kunst und Kultur, Glücksspiel. Bei letzterem schlägt sich offensichtlich die relativ hohe Arbeitsintensität nieder, denn die Auswirkungen für die gesamtwirtschaftliche Bruttowertschöpfung sind hier relativ gering. Dasselbe gilt für das bezogen auf die Beschäftigungswirkung am dritten Rang liegende Gastgewerbe. Vergleichsweise hohe Beschäftigungseffekte haben daneben die Branchen, die auch schon hohe volkswirtschaftliche Verluste zur Folge hatten, also Nahrungs- und Futtermittel, Getränke, Tabakerzeugnisse, der Einzelhandel (ohne Handel mit Kraftfahrzeugen), Landverkehrs- und Transportleistungen in Rohrfernleitungen sowie die Erbringung von Finanzdienstleistungen.

## Fazit

In diesem Beitrag werden die Branchen identifiziert, die aus ökonomischer Sicht besonders systemrelevant sind. Als ökonomisch systemrelevant bezeichnen wir Branchen, die aufgrund der dort produzierten Vorleistungen besonders eng über die Wertschöpfungsketten mit der übrigen Wirtschaft verflochten sind, sodass ein Produktionsstopp in diesen Bereichen die Gesamtwirtschaft besonders stark in Mitleidenschaft zieht. Gerade in der jetzigen Krise im Zuge der COVID-19-Pandemie könnte diese Analyse von besonderer Relevanz in Bezug auf mögliche Konjunkturprogramme sein. Zur Identifikation wird in einem ersten Schritt die Input-Output-Tabelle des Statistischen Bundesamts ausgewertet und in einem zweiten Schritt die volkswirtschaftlichen Kreislaufeffekte (Lohn-Einkommen-Konsum, Investitionsentscheidungen) eines Produktionsrückgangs der identifizierten

Branchen mit Hilfe eines makroökonomischen Modells analysiert.

Es zeigt sich, dass eine ganze Reihe von Branchen in der Wirtschaft sehr eng verflochten ist und somit eine hohe ökonomische Systemrelevanz besitzt. So gibt es Branchen, bei denen Produktionsausfälle über Input-Output-Verflechtungen bzw. über Kreislaufwirkungen zu massiven Nachfrageausfällen führen. Der in der Öffentlichkeit oft genannte Fahrzeugbau gehört zwar auch dazu, allerdings nimmt er nicht die Spitzenposition ein, die aufgrund der öffentlichen Wahrnehmung zu erwarten gewesen wäre. Vielmehr sind es der Handel und die Logistik, die eine sehr hohe Bedeutung für die Volkswirtschaft haben. Diese Branchen, die mit der Bündelung und Verteilung von Warenströmen Geld verdienen, sind für die Zirkulation der Güter von Produzenten zu Endverbrauchern zuständig.

Daneben gibt es Branchen, wie beispielsweise Metallerzeugnisse, Maschinen und chemische Erzeugnisse, bei denen Produktionsausfälle zu besonders großen Lieferschwierigkeiten an andere Branchen führen. Das kann bei kurzfristig unbedingt nötigen Lieferungen gravierende Probleme nach sich ziehen. Andere Vorleistungen, wie beispielsweise Dienstleistungen der Rechts-, Steuer- und Unternehmensberatung, die Wach-, Sicherheitsdienstleistungen und wirtschaftliche Dienstleistungen oder die sonstigen Unternehmensdienstleistungen sind zwar mittelbar ebenfalls bedeutend und haben damit eine hohe Systemrelevanz, würden aber nicht unmittelbar zu einem Produktionsstopp führen.

Bezogen auf den Arbeitsmarkt sind es die sonstigen Unternehmensdienstleistungen, die bei einem Produktionsausfall die größten negativen Beschäftigungseffekte nach sich ziehen, dicht gefolgt von den Dienstleistungen der Kunst, der Kultur und des Glücksspiels. Bei letzterem schlägt sich offensichtlich die relativ hohe Arbeitsintensität nieder, sind hier doch die Auswirkungen für die gesamtwirtschaftliche Bruttowertschöpfung relativ gering. Dasselbe gilt für das bezogen auf die Beschäftigungswirkung am dritten Rang liegende Gastgewerbe. Vergleichsweise hohe Beschäftigungseffekte haben daneben auch die Branchen, in denen ein Produktionsstopp schon hohe gesamtwirtschaftliche Verluste bei der Bruttowertschöpfung zur Folge hatte, also die Branchen Nahrungs- und Futtermittel, Getränke, Tabakerzeugnisse, der Einzelhandel (ohne Handel mit Kraftfahrzeugen), die Landverkehrs- und Transportleistungen in Rohrfernleitungen sowie die Erbringung von Finanzdienstleistungen.

Gerade das Gastgewerbe, die Dienstleistungen der Kunst, der Kultur und des Glücksspiels und der Einzelhandel (ohne Handel mit Kraftfahrzeugen) mit Ausnahme des Lebensmitteleinzelhandels waren und sind in Teilen immer noch von der aktuellen COVID-19-Pandemie besonders gefordert. Bei möglichen weiteren Konjunkturprogrammen wird es darauf ankommen, gegebene fiskalische Mittel möglichst effektiv einzusetzen – sodass sich möglichst umfassende positive Folgewirkungen in der Gesamtwirtschaft ergeben. Dies ist immer dann gegeben, wenn durch eine staatliche Maßnahmen Einkommen in großem Umfang entstehen. Das wäre besonders bei Maßnahmen in ökonomisch systemrelevanten Branchen entsprechend der vorliegenden Analyse der Fall, weil die Förderung der Wertschöpfung in einer Branche über Lieferverflechtungen und Kreislaufzusammenhänge auch die Wertschöpfung über diese Branche hinaus besonders positiv beeinflusst. In ähnlicher Weise argumentieren auch Merkl und Weber (2020) und Weber (2020), dass eine gezielte finanzielle Unterstützung von Neueinstellungen expansive Wirkungen in Wirtschaft und Arbeitsmarkt deutlich effektiver erzielen könnte als ein breit gestreutes Ausgabenprogramm.

**Title:** ***Which Industries Are Economically Relevant to the System?***

**JEL Classification:** *C67, D57, E17.*
